# Surgical Skills Day: Bridging the Gap

**DOI:** 10.7759/cureus.8131

**Published:** 2020-05-15

**Authors:** Keng Siang Lee, Sebastian Priest, Joshua J Wellington, Toluwaniyin Owoso, Leyln Osei Atiemo, Ameen Mardanpour, Zachary Craft, Natalie Blencowe, Robert J Hinchliffe

**Affiliations:** 1 Medicine, Bristol Medical School, University of Bristol, Bristol, GBR; 2 Surgery, University Hospitals Bristol and Weston NHS Foundation Trust, Bristol, GBR; 3 Vascular Surgery, North Bristol NHS Trust, Bristol, GBR

**Keywords:** surgical skills day, surgery, undergraduate training, medical education, foundation programme, united kingdom, clinical skills

## Abstract

Background

The General Medical Council (GMC) requires all newly qualified doctors to be competent in certain surgical skills, including the provision of basic wound closure. Yet there is a profound lack of undergraduate competence in, and exposure to, basic surgical skills such as wound closure. The Surgical Skills Day (SSD) aimed to provide medical students with additional skills training.

Methods

Student self-assessment and instructors’ assessment forms were completed prior to and following a workshop on basic wound closure skills. Paired t-tests was used to statistically compare the two pre and post-instruction data sets.

Results

A total of 46 students attended the SSD; 29 consented to the skills assessment. 100% (n = 29) self-reported improved competency in at least one of the skills following tuition (p < 0.001). Instructors’ assessment agreed that 100% (n = 29) of students improved in at least one of the skills assessed (p < 0.001). 100% of the attendees agreed that additional practical surgical skills should be incorporated into the undergraduate curriculum. 64% (n = 21) of students also confirmed that they were more likely to pursue a career in surgery following the SSD.

Conclusion

Current clinical teaching in basic suturing is unsuitable for long term retention. SSDs can improve skills acquisition and elevate student confidence. This data builds on our previous work by documenting the high efficacy in skills acquisition as a result of SSD tuition. We recommend that SSDs be integrated into medical school curricula in order to address shortcomings in current undergraduate programmes.

## Introduction

Medical students across the United Kingdom (UK) report poor satisfaction with surgical teaching and inadequate preparation for surgical rotations during the Foundation Programme (FP) [1,2]. There is a lack of undergraduate experience in surgical settings, primarily due to a shift in emphasis towards acquiring other attributes, such as communication skills, and fulfilment of governmental mandates on recruiting general practitioners [3,4]. The General Medical Council (GMC) requires all newly qualified doctors to be competent in certain surgical skills, including the provision of basic wound closure [5]. However, the reduced focus on surgical skills in undergraduate curricula may leave newly qualified doctors at risk of being unable to successfully perform basic surgical tasks safely and confidently under guidance [6]. This is despite the GMC requiring all newly qualified doctors to be competent in technical skills/procedures [5]. Critically, this highlights the growing demand and need for surgical skills workshops, which have been shown to stimulate interest in surgery in medical student cohorts, and provide an opportunity to practice basic surgical skills [7,8].

The Surgical Skills Day (SSD), delivered by the University of Bristol Surgical Society (SCRUBS) is a student-led initiative that aims to provide students with the opportunity to: access additional skills training, further explore any career interest in surgery and gain access to surgeons in training for networking opportunities. This article aims to build on our previous reporting of the SSD, examining primarily whether teaching workshops were useful in terms of skills acquisition [9]. Our previous findings revealed that undergraduate surgical teaching is deficient and that the SSD improved engagement in practical skills and heightened enthusiasm in surgery as a career. In this article, we focus on basic suturing, as this is a GMC mandated skill required of all junior doctors. The aims of this article are to identify the need for incorporating more surgical skills training into medical undergraduate curricula; assess the utility and efficacy of tutoring on the SSD; and provide recommendations to maximise the practicality, efficacy, and integration of future faculty-led SSDs.

## Materials and methods

The SSD, held on November 10, 2019, is a one-day practical course primarily aimed at UK medical students, offering a diverse range of one-hour workshops. These workshops included laparoscopic simulation, dynamic hip screw placement using model femurs, burr hole drilling on model skulls, tracheostomy, porcine aortic re-anastomosis, tendon repair, and suturing using porcine models. Students were allocated in groups of nine to 12 to a set circuit including these workshop stations. Each workshop was assigned two instructors who were UK surgeons of various training grades.

Students were only assessed on the ‘suturing using porcine models’ station as these skills were directly applicable to the competencies mandated by the GMC for foundation doctors [5]. These competencies were assessed by instructors and using self-reported questionnaires. Competencies were evaluated prior to and following instruction. This allowed the authors to map progression following instructor-led tuition.

The suturing workshop taught and assessed the following seven skills mandated by the GMC for foundation doctors: mounting the needle, simple interrupted suture, vertical mattress suture, horizontal mattress suture, subcuticular suture, suture removal and the safe disposal of sharps.

Instructors assessed a student’s competency in these seven skills by observing them performing these skills. Students were then scored on a binary ‘can perform under guidance/cannot perform under guidance’ (Table 1). This is in line with the GMC definition of foundation level competency: ‘safe to practice under direct supervision’ [5].

**Table 1:** Pre and post instructional form supplied to instructors for their assessment of students. Boxes were selected accordingly.

Skill	Can perform under guidance	Cannot perform under guidance
Mounting the needle		
Simple Interrupted suture		
Vertical Mattress suture		
Horizontal Mattress suture		
Subcuticular suture		
Suture removal		
Safe disposal of sharps		

Students were asked to evaluate their competency in these skills by selecting one of the following categories for each skill: ‘Cannot/Have never performed’, ‘Can perform with guidance’, ‘Can perform independently but require guidance at times’ and ‘Can perform independently’ (Table 2).

**Table 2:** Pre and post instructional form supplied to students for their self-assessment. Boxes were selected accordingly.

Skill	Cannot/Have Never Performed	Can Perform with guidance	Can perform independently but require guidance at times	Can perform independently
Mounting the needle				
Simple Interrupted suture				
Vertical Mattress suture				
Horizontal Mattress suture				
Subcuticular suture				
Suture removal				
Safe disposal of sharps				

The assessments performed prior to the workshop were carried out following instruction using identical methods to ensure comparability. Students were supplied with extra categories in the self-assessment to help them map their progression following tuition. Instructors’ assessments were limited to binary choices to streamline the process of assessing competency. However, for comparability, it was determined that categories above and including ‘can perform with guidance’ for the student-reported outcomes would be classified as competent in line with the GMC definition provided.

All competency data was anonymised and evaluated using Sigma Plot (Jandel Scientific, Chicago, IL). Statistical analysis employed paired t-tests. Power was calculated to ensure an adequate numerical study population (α = 0.05, Power = 80%). A sub-group analysis of students in the clinical phase of their training (Year 3 and above) was also performed as these students should have all received basic suturing instruction as part of the curriculum.

Students were also supplied with a post-day evaluation form that was made available to them 24 hours after the event. Responses were encouraged in exchange for certificates of attendance for surgical portfolios. This evaluation form remained identical to that employed in our previous research, including the question ‘Would you like to see more practical surgical skills incorporated into medical curriculum?’ [9].

## Results

Demographic overview

Medical students from across the UK were recruited for the SSD using advertisement on social media via UK undergraduate surgical societies and targeted emailing via membership lists. The SSD was open to medical students of all grades. In total, 46 students attended the 2019 SSD, all of whom attended Bristol Medical School. Of these, 29 (63%) were female. 24 (52%) of these students were in the clinical phase of their training which was defined as the third year of medical school and above. Students were recruited for the skills assessment on an ‘opt-in’ basis. Altogether 29 of the 46 student attendees (63%) opted for the skills assessment. The power calculations using sigma plot also demonstrated that our study was adequately powered (80%).

Self-reported competencies

To examine competency in the self-reported data, which was defined as ‘can perform with guidance’ and above, we assigned binary values to each category (Table 3). A value of 0 indicated a lack of competency and a value of 1 indicated competency. Across the seven skills assessed this gave a maximum value of 7. Prior to instruction, students reported an average competency score of 3.21 (± 0.58). Following instruction, students reported an average competency score of 6.69 (± 0.10). This demonstrates a self-reported improvement of 3.48 (±0.54) (p < 0.001) in average values.

**Table 3:** Overall results documenting the number of students competent, as per the self-reported questionnaire and instructors’ assessment. The table demonstrates the numbers of competent students before and following instruction, with the numbers of students becoming following tuition competent displayed in the final two columns.

	Self-Reported Competence (Prior to Instruction)	Instructor Assessment (Prior to Instruction)	Self-Reported (After Instruction)	Instructor Assessment (After Instruction)	Number of students improving (Self-Reported)	Number of students improving (Instructor Assessment)
Mounting the needle	16 (55%)	11 (38%)	29 (100%)	29 (100%)	13 (45%)	18 (62%)
Simple Interrupted suture	14 (48%)	8 (28%)	29 (100%)	28 (97%)	15 (52%)	20 (69%)
Vertical Mattress suture	10 (34%)	1 (3%)	29 (100%)	22 (76%)	19 (66%)	21 (72%)
Horizontal Mattress suture	12 (41%)	1 (3%)	28 (97%)	22 (76%)	16 (55%)	21 (72%)
Subcuticular suture	9 (31%)	1 (3%)	22 (76%)	8 (28%)	13 (45%)	7 (24%)
Suture removal	16 (55%)	14 (48%)	28 (97%)	28 (97%)	12 (41%)	14 (48%)
Safe disposal of sharps	16 (55%)	16 (55%)	29 (100%)	29 (100%)	13 (45%)	13 (45%)

Instructor assessment

The instructors examined the competency of individual suturing skills in line with the GMC definition of competency. Students were assessed before and after instruction (Table 3). Instructors assigned binary values to students, using 1 to indicate competency and 0 to indicate incompetency. Prior to instruction, the average competency score was 1.80 (± 0.33) and following instruction, this rose to 5.72 (± 0.17), which represents an improvement of 3.92 (±0.40) (p < 0.001) in mean scores.

Post-day feedback

Further to the quantitative data collected, the 29 students were asked ‘Would you like to see more practical surgical skills incorporated into medical curriculum?’. The post-day feedback concluded that all 29 (100%) agreed that additional practical surgical skills should be incorporated into the undergraduate curriculum. Additionally, 21 (72%) students confirmed that they were more likely to pursue a career in surgery following the SSD while 11 (38%) and one (3%) were unsure and not more likely to, respectively.

Sub-group analysis of clinical phase students

Nineteen of the 24 clinical phase students (79%) had consented for skills assessment. A sub-group analysis of these 19 clinical phase students was also performed as these students should have all received basic suturing instruction as part of the curriculum (Table 4). Prior to instruction, the average self-reported competency score was 3.11 (± 0.70), and following instruction, this rose to 6.63 (± 0.14), which represents an improvement of 3.53 (±0.64) (p < 0.001) in mean scores. The average instructor-assessed competency score prior to tuition was 2.00 (± 0.45) and following instruction, this rose to 5.74 (± 0.24), which represents an improvement of 3.74 (±0.55) (p < 0.001) in mean scores.

**Table 4:** Documenting the number of clinical students competent, as per the self-reported questionnaire and instructors’ assessment. The table demonstrates the numbers of competent students before and following instruction, with the numbers of students becoming competent after tuition displayed in the final two columns.

	Prior to instruction		Following instruction		Number of students improving	
Components of basic suturing assessed	Student self-reported data	Instructor assessment	Student self-reported data	Instructor assessment	Student self-reported data	Instructor assessment
Mounting the needle	11 (58%)	10 (53%)	19 (100%)	19 (100%)	8 (42%)	9 (47%)
Simple interrupted suture	9 (47%)	7 (37%)	19 (100%)	18 (95%)	10 (53%)	11 (58%)
Vertical mattress suture	6 (32%)	0 (0%)	19 (100%)	14 (74%)	13 (68%)	14 (74%)
Horizontal mattress suture	8 (42%)	0 (0%)	18 (95%)	14 (74%)	10 (53%)	14 (74%)
Subcuticular suture	6 (32%)	1 (5%)	14 (74%)	7 (37%)	8 (42%)	6 (32%)
Suture removal	11 (58%)	9 (47%)	18 (95%)	18 (95%)	7 (37%)	9 (47%)
Safe disposal of sharps	11 (58%)	11 (58%)	19 (100%)	19 (100%)	8 (42%)	8 (42%)

## Discussion

The skills gap

The GMC lists wound care and basic wound closure among the core practical skills and procedures that newly qualified doctors must be able to safely complete under direct supervision [5].

A national review of surgical skills training in UK medical schools highlights a significant deficiency in basic procedural skills training, including wound suturing. Only 24.7% of medical schools provided suturing training while the majority of skills acquisition was pioneered by extracurricular student-led surgical societies, 64.5% of which provided suturing training [6]. Despite the absence of national statistics, the apparent lack of skills training in UK medical schools has been further supported in recent studies [2]. The failure to provide undergraduates with structured teaching that meets the GMC graduate outcomes has led to more newly-qualified doctors being unable to safely perform this practical procedure, and hence being unprepared for some aspects of clinical practice [10,11]. This demonstrates the necessity for greater incorporation of surgical skills into undergraduate training.

We appreciate that the seven skills examined during the skills assessment varied greatly in difficulty (e.g. mounting the needle vs subcuticular suturing), and are of varying importance to the average medical students and newly qualified foundation doctor. Hence, these individual skills were further assessed to reflect these differences.

Skills of the lowest perceived difficulty such as ‘mounting the needle’, ‘suture removal’ and the ‘safe disposal of sharps’ were shown to have the highest pre-workshop competency levels, with 38%, 48% and 55% of the students being deemed competent by instructors, respectively. Conversely, skills perceived to be more challenging such as the ‘horizontal mattress suture’ and ‘subcuticular suture’ demonstrated low levels of competency with only 3% of students displaying proficiency.

Nonetheless, despite the contrast in skill difficulty, the study cohort still demonstrated significant (p < 0.001) improvements in all skills that were assessed following instruction. All students demonstrated competency in ‘mounting the needle’ and the ‘safe disposal of sharps’, while 76% and 28% of students were proficient in the ‘horizontal mattress suture’ and ‘subcuticular suture’, as assessed by the instructors respectively. This further demonstrates that an hour period of intense tuition can significantly improve student competencies in these suturing skills. However, some individual students may likely require further support and tuition if they are to attain competency in skills of greater difficulty.

Sub-group analysis of clinical-phase medical students determined that they were largely deficient in multiple suturing skills prior to SSD instruction. This not only highlights that current skills training delivered at the undergraduate level is unsuitable for long-term retention, but that frequent application of these skills is required to maintain competency. The significant rates of improvement in competencies for the students also highlight that one hour of SSD tuition can address these deficiencies in the current curriculum. Therefore, we recommend that students receive frequent, structured training in basic surgical skills, similar to that delivered on the SSD. This would address current undergraduate deficiencies in competency and ensure long-term skills retention.

Student demand

The demand for the incorporation of SSDs into the medical curriculum has also been echoed by all our 29 students. This response could arguably be influenced by the fact that most students attending a SSD will likely have an existing interest in surgery. However, only 21 (72%) of students confirmed that they were more likely to pursue a career in surgery following the SSD while 11 (38%) were unsure. This shows that regardless of specialist interests, medical students are aware of the deficiency in their basic surgical skills training in their undergraduate curricula and agree that SSDs could be an effective way to bridge this gap. 

**Figure 1:**
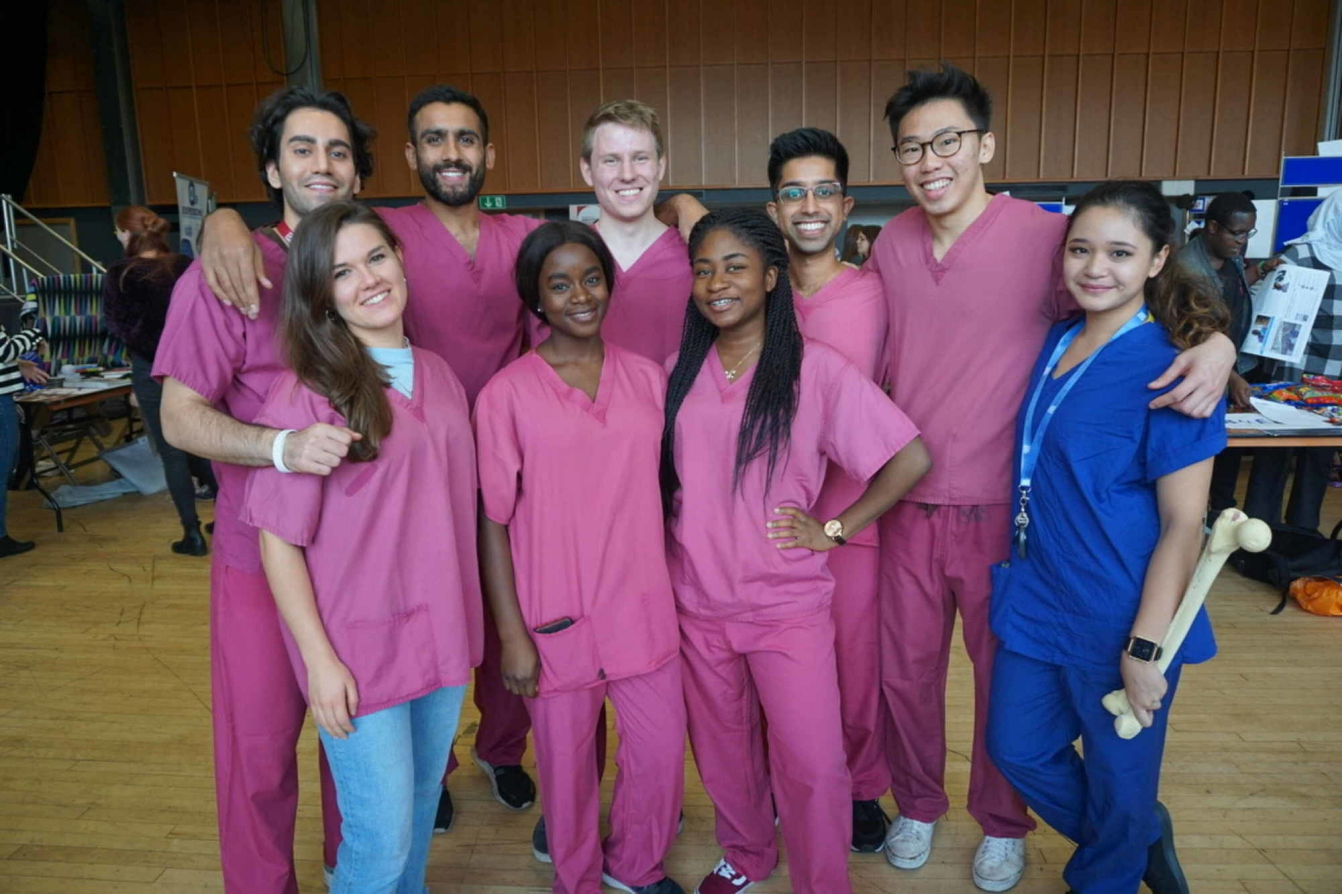
The University of Bristol Surgical Society (SCRUBS) team.

On our SSD, students are only tutored for one hour. However, it is commonly accepted that for long term retention of skills, frequent application is required. A student-led surgical society will not have the capacity to ensure that all students have access to training and leave medical school possessing GMC mandated competencies. Nonetheless, the data on skills acquisition, as displayed above, does acknowledge that one hour of trainee-led teaching results in significant (P<0.001) improvements across basic wound care skills. Consequently, it is strongly encouraged that the SSD training model is uniformly integrated and revisited in the undergraduate curriculum on multiple occasions so as to provide access to all students in the cohort and ensure skills retention. Figure 1 represents the SCRUBS team that led this SSD.

Feasibility

Incorporating the SSD format into medical school curricula is not without challenges, however, it remains an attainable goal. Here, recommendations for implementation are provided. Firstly, course directors must decide where in the curriculum they wish to place SSDs. Secondly, a teaching specification of surgical skills must be created. Thereafter, responsibility for organising SSDs must be devolved to regional teaching academies. In doing so, the number of student attendees (and consequently teachers) remains low and manageable. Additionally, academies can utilise their teaching facilities and the expertise of dedicated clinical teaching fellows.

Limitations

Certain limitations of this study must also be acknowledged. Firstly, our participants likely represent a select group of students who are already surgical enthusiasts to begin with, which could have inflated our positive feedback gathered. Further studies are needed to see if such favourable results can be replicated, and also to ensure if they are generalisable. A random tutorial group which may include those not interested in surgery, could be invited to our next workshop, to assess the whether our SSD could benefit all medical students equally regardless of specialty interest as basic suturing is a required skill.

Secondly, in our study, competency was given a binary outcome. This was in accordance with how medical school competencies are assessed. We were hence unable to measure the level of competency, but the focus of our study was only to determine whether medical students could perform at the level required of a foundation doctor.

In addition, we recognise that our study did not attempt to ascertain whether students could retain their knowledge and skills until the foundation programme. We will attempt a retention study by inviting the same 29 students who consented to assessment to the SSD of the next academic year of 2020/2021, in order to demonstrate the same trend of student improvement to be deemed truly useful.

For a programme such as our SSD to meet GMC guidelines, important additions to the teaching content would need to be made. This includes incorporating an aspect of the training session addressing wound cleaning and administration of a local anaesthetic. These skills were not addressed as we deemed that including teaching on these aspects within the station would have compromised the time spent on core suturing techniques. Nonetheless, this restates the case that basic surgical skills training should be delivered as part of a structured medical school curriculum, where time restrictions may be of less concern and skills can be taught in greater depth.

## Conclusions

In conclusion, medical students largely lack basic surgical skills mandated by the GMC. Furthermore, teaching that has been provided to clinical years students is ineffective at providing long term retention. The SSD student-led initiative can improve skills acquisition, elevate student confidence, and provide a valuable ‘taster’ of a potential career in surgery. We recommend, as in line with our previous work, that the SSD or similar surgical training events be integrated into medical school curricula to address skills deficits in current undergraduate programmes.
